# Regulation of Neural Differentiation of ADMSCs using Graphene‐Mediated Wireless‐Localized Electrical Signals Driven by Electromagnetic Induction

**DOI:** 10.1002/advs.202104424

**Published:** 2022-02-12

**Authors:** Zhijie Guo, Chunhui Sun, Hongru Yang, Haoyang Gao, Na Liang, Jian Wang, Shuang Hu, Na Ren, Jinbo Pang, Jingang Wang, Ning Meng, Lin Han, Hong Liu

**Affiliations:** ^1^ Collaborative Innovation Center of Technology and Equipment for Biological Diagnosis and Therapy in Universities of Shandong Institute for Advanced Interdisciplinary Research (iAIR) University of Jinan Jinan 250022 P. R. China; ^2^ State Key Laboratory of Crystal Materials Shandong University Jinan Shandong 250100 P. R. China; ^3^ School of Biological Science and Technology University of Jinan Jinan Shandong 250022 P. R. China; ^4^ Institute of Marine Science and Technology Shandong University Qingdao Shandong 266200 P. R. China

**Keywords:** adipose‐derived mesenchymal stem cells, electromagnetic induction, graphene film, magneto‐electric biomaterial, neural differentiation

## Abstract

Although adipose‐derived mesenchymal stem cells (ADMSCs) isolated from patients’ fat are considered as the most important autologous stem cells for tissue repair, significant difficulties in the neural differentiation of ADMSCs still impede stem cell therapy for neurodegenerative diseases. Herein, a wireless‐electrical stimulation method is proposed to direct the neural differentiation of ADMSCs based on the electromagnetic effect using a graphene film as a conductive scaffold. By placing a rotating magnet on the top of a culture system without any inducer, the ADMSCs cultured on graphene differentiate into functional neurons within 15 days. As a conductive biodegradable nanomaterial, graphene film acts as a wireless electrical signal generator driven by the electromagnetic induction, and millivolt‐level voltage generated in situ provokes ADMSCs to differentiate into neurons, proved by morphological variation, extremely high levels of neuron‐specific genes, and proteins. Most importantly, Ca^2+^ intracellular influx is observed in these ADMSC‐derived neurons once exposure to neurotransmitters, indicating that these cells are functional neurons. This research enhances stem cell therapy for neurodegenerative diseases using autologous ADMSCs and overcomes the lack of neural stem cells. This nanostructure‐mediated physical‐signal simulation method is inexpensive, safe, and localized, and has a significant impact on neural regeneration.

## Graphical abstract

This novel stimulation method does not rely on the function of neural‐inducing biological factors, but only utilizes the conducting properties of a scaffold material (graphene) to convert external magnetic fields into electrical fields based on electromagnetic induction. The resultant wireless electric stimulation inspires the adipose‐derived mesenchymal stem cells to differentiate into functional neurons to transmit nerve impulses.

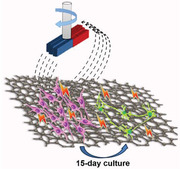

## 1 Introduction

Neurodegenerative diseases occur in the central, peripheral, and autonomic nervous systems and are characterized by sensory, motor, conscious, and autonomic dysfunctions.^[^ ^1^ ^]^ The main pathology of neurological diseases is the progressive loss of functional neurons, which fail to translate nervous impulses and cause neurodegenerative diseases.^[^ ^2^ ^]^ Neural regeneration is a promising method for neurodegenerative disease therapy, which is based on stem cells such as embryonic stem cells (ESCs), mesenchymal stem cells (MSCs), neural stem cells (NSCs), and induced pluripotent stem cells (iPSCs). However, undifferentiated inner mass cells of a human embryo or viral‐mediated transfections are required to harvest ESCs^[^ ^3^ ^]^ or iPSCs,^[^ ^4^ ^]^ which is a significant obstacle to clinical applications, considering ethical and security challenges. Quiescent NSCs reside in the subventricular zone of the forebrain and subgranular zone of the hippocampus^[^ ^5^ ^]^ and have been reported to be activated by the stimulation of neurotrophic factors to participate in neurogenesis.^[^ ^6^ ^]^ Nonetheless, a recent study revealed that NSCs in the hippocampus and neurogenesis are extremely rare in adult humans,^[^ ^7^ ^]^ indicating the limited application of endogenous NSCs in neural repair. The transplantation of allogeneic stem cells has provided a new route for the treatment of neurodegenerative diseases. However, immunological rejection cannot be ignored.

Compared with NSCs, which have limitations such as limited sources and difficulty of extraction, MSCs have a wide range of sources, including the bone marrow, umbilical cord, cord blood, placenta, fat, and other tissues. Particularly, adipose‐derived mesenchymal stem cells (ADMSCs), isolated from subcutaneous fat through liposuction with minimal trauma, are easier to extract and expand faster. Their multidirectional differentiation capability and the ability to secrete various biologically active factors make them candidates for various applications in autologous stem cell transplantation. In addition, the potential of ADMSCs in neural differentiation has been recognized^[^ ^8^ ^]^ and these ADMSC‐derived nerve cells have been demonstrated to promote nerve repair.^[^ ^9^ ^]^ More importantly, ADMSCs exhibit low immunogenicity and tumorigenicity. Clinical trials have proved that the application of ADMSCs in the repair of organ damage, such as for the heart, rectum, and breast, is safe, effective, and feasible.^[^ ^10^ ^]^ ADMSCs are widely accepted ideal seed cells in neural tissue engineering and are excellent candidates for clinical applications.

Efforts have been conducted to aid the transformation of MSCs into nerve cells, including a mixture of growth factors,^[^ ^11^ ^]^ supplementation with chemical agents,^[^ ^12^ ^]^ and over‐expression of related factors by gene transfection.^[^ ^13^ ^]^ Among them, cytokine administration is restricted from bench‐to‐bedside applications owing to its expansiveness, short half‐life period, and lack of noninvasive delivery methods.^[^ ^14^ ^]^ Chemical stimulation is also challenging because of undefined neuron functions, and neuron‐like morphological changes may be associated with cell shrinkage because of cellular toxicity.^[^ ^15^ ^]^ For genetic manipulation, the low transfection efficiency as well as biosafety, such as the risk of proto‐oncogene activation by viral vectors, should be considered carefully before clinical applications.^[^ ^16^ ^]^ Note that these MSC‐derived nerve cells may not effectively integrate into lesion cavities or fuse with the original neural network, although the above‐mentioned methods of inducing the neural differentiation of MSCs can be conducted in vitro. Furthermore, another challenge for these transplanted cells is uncontrollable destinations, including indeterminate differentiation fate and random migration. A nerve scaffold must be implanted to support and immobilize seed cells in the appointed area to guide and promote axon growth, particularly for large nerve defects.

As one of the three parts of neural tissue engineering, scaffold materials with good biocompatibility provide spatial support to cells and their micro/nanostructures,^[^ ^17^ ^]^ and mediated physical signals^[^ ^18^ ^]^ can contribute significantly to inducing stem cell differentiation. Recently, due to the electrical activity of neurons, the combination of conductive biomaterials with electrical stimulation has attracted much interest in the prospect of neural differentiation. To achieve electrical stimulation, researchers generally select conductive substrates for stem cell culture and use a pair of wires to achieve pulsed electrical stimulation. Reduced graphene oxide microfibers have been designed using a triboelectric nanogenerator to promote the neural differentiation of MSCs.^[^ ^19^ ^]^ However, the electrical signals introduced by the external wires with electrical signal‐generating apparatus are not suitable for clinical use and will cause inconvenience or secondary damage to the patients. The realization of in situ electrical signal inputs without wire implantation is an urgent problem.

To solve these problems, we propose a concept of stimulating stem cells using nanostructure‐mediated electrical signals. Based on the phenomenon of electromagnetic induction and the working principle of an electric generator, a conductor generates an induced current when it cuts magnetic induction lines. Thus, we selected graphene as a nerve scaffold as it is a promising conductor^[^ ^20^ ^]^ and a 2D material with a single atomic layer composed of sp^2^‐hybridized carbon atoms, relying on covalent bonds.^[^ ^21^ ^]^ Graphene‐based materials have been applied in the fields of biomedicine, including biosensing,^[^ ^22^ ^]^ cancer treatment,^[^ ^23^ ^]^ disease diagnosis,^[^ ^24^ ^]^ and drug delivery.^[^ ^25^ ^]^ The biocompatibility of graphene has been confirmed, and it can be degraded in vivo using human neutrophil peroxidase,^[^ ^26^ ^]^ and the degradation products have no toxic side effects,^[^ ^27^ ^]^ indicating its biosafety and prospects for clinical application. In tissue engineering, the research on graphene‐based materials has primarily focused on bone and neural regeneration.^[^ ^28^ ^]^ Graphene‐based materials are expected to be a new type of neural interface material in the future.^[^ ^29^ ^]^ Several configurations of graphene scaffolds have been studied and prove that graphene substrates can promote the neural differentiation of NSCs,^[^ ^30^ ^]^ iPSCs,^[^ ^31^ ^]^ ESCs,^[^ ^32^ ^]^ and MSCs.^[^ ^33^ ^]^ However, the positive effect of graphene on neuro‐differentiation is achieved by adding differentiation inducers or depends on the topological structure of graphene. Whether wireless electrical stimulation mediated by graphene‐based electromagnetic induction can induce the neural differentiation of stem cells is unclear and has not been reported.

To prove our hypothesis that graphene generates electric signals by cutting magnetic induction lines and this induced micro‐current may promote the neural differentiation of ADMSCs, in this study, we synthesize graphene films through chemical vapor deposition. The model of an immobile graphene film under a rotating magnetic field (MF) is adopted to imitate electromagnetic induction. After the confirmation of the electricity‐generation performance and biocompatibility, ADMSCs cultured on graphene film are stimulated using a rotating MF, and the potential of neural differentiation is further investigated. The results demonstrate that the electrical signal derived from the graphene film driven by a rotating MF, without any supplement of inducers, is sufficient to trigger the differentiation of ADMSCs into functional neurons. Furthermore, the electromagnetic‐induction‐driven electrical signal regulates ADMSCs into directly differentiating into neurons and restricts neuroglia. This study reveals an interesting result that a rotating MF can induce MSCs on graphene films to directly differentiate into functional neurons without any growth factor. These findings provide a new strategy for nerve repair through wireless and localized electric stimulation driven by magneto‐electric biomaterials.

## 2 Results and Discussion

### 2.1 Characterization of Graphene Films

**Figure 1:**
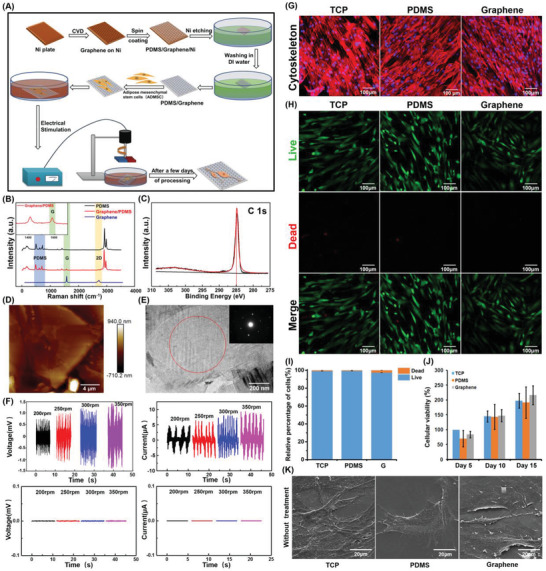
Characterization and cell compatibility of graphene film. A) Schematic fabrication of graphene film. B) Raman spectrum of graphene. The inset image is the spectrographic magnification of graphene/PDMS from 1300 to 1700 cm^−1^. C) High‐resolution C (1s) XPS spectra of graphene. D) AFM image of graphene. E) TEM image of the graphene surface and upper right insert was the electron diffraction pattern of the area. F) Different current or voltage intensity changes caused by different MF strengths in graphene (the upper panels) and vehicle control (PDMS, the lower panels). G) Image of cytoskeleton staining and H) live/dead staining in different samples without MF treatment. The live cells were stained green, and the dead cells were stained red. I) Quantification of surviving ADMSCs after culturing in different samples shown in (H). J) Relative cellular viabilities in different groups were analyzed by CCK‐8 staining. All data represent the mean ± standard deviation (*n* = 3). K) SEM images of ADMSCs cultured on different substrates for 3 days.

The graphene film was synthesized on a nickel plate via chemical vapor deposition (**Figure** **1**A). To ensure the integrality and handleability of the graphene film during cell experiments, graphene was transferred onto poly(dimethyl siloxane) (PDMS) and was termed graphene/PDMS (Figure 1A). PDMS is a biocompatible polymer with superior elasticity and flexibility and has been adopted as a medical implant because of its nontoxicity to cells and high permeability to gases.^[^ ^34^ ^]^ The graphene film was characterized using Raman spectroscopy, X‐ray photoelectron spectroscopy (XPS), atomic force microscopy (AFM), transmission electron microscopy (TEM), and scanning electron microscopy (SEM). As shown in the Raman spectra in Figure 1B and the Raman mapping image in Figure S1, Supporting Information, graphene/PDMS exhibited both the characteristic peaks of native PDMS and graphitic carbon, i.e., the G peak (≈1580 cm^−1^) of sp^2^ hybridization^[^ ^35^ ^]^ and 2D peaks located at 2686 cm^−1^.^[^ ^36^ ^]^ The number of graphene layers in a film can be estimated using the ratio of the intensities of the G and 2D peaks^[^ ^37^ ^]^ and the intensity of 2D peaks is negatively correlated with graphene layers.^[^ ^38^ ^]^ The ratio *I*_2D_:*I*_G_ < 1 represents multilayer graphene, while *I*_2D_:*I*_G_ > 1 represents few/single‐layer graphene.^[^ ^38^, ^39^ ^]^ The Raman spectra in Figure 1B and Figure S2 in the Supporting Information suggested that the synthesized graphene film was constructed using multilayer graphene sheets. In addition, the detect‐related D peak (in the range 1300–1370 cm^−1^)^[^ ^40^ ^]^ was not observed in the graphene/PDMS, indicating that the obtained graphene film was of high quality. XPS was performed to characterize the surface chemistry of the graphene films. As shown in Figure 1C, a noticeable peak near 284.6 eV was detected in the C 1s XPS spectrum of pure graphene, which corresponded to the nonoxygenated ring C and emerged from sp^2^‐hybridized carbon atoms (C═C bond).^[^ ^41^ ^]^ In addition, the SEM image in Figure S1 in the Supporting Information shows that the surface of the graphene film was relatively smooth, and the folded strata of the film revealed a tight contact between the graphene sheet and substrate. To ensure the consistency of the graphene film and reduce the effect of the substrate thickness on the induced electric potential, we synthesized all the graphene samples under the same reaction conditions, including the gas flow ratio, reaction temperature, and reaction time. Different batches of graphene films exhibited similar *I*_2D_:*I*_G_ ratios, indicating that the number of layers was approximately the same (Figure S2D, Supporting Information). Moreover, the typical electron diffraction pattern exhibited by TEM (Figure 1E) also revealed the electron diffraction pattern of multilayer graphene, suggesting a good crystalline of graphene. The TEM images of the cross‐section and edges of graphene were displayed in revised Figure S2 in the Supporting Information. A typical image of multilayer graphene sheet was shown in Figure S2A in the Supporting Information, where graphene interface with single layer could be observed. In addition, a portion of folded graphene existed on the surface, and the formation of a few layers of graphene was discovered at the folded edge rather than a monolayer. Detailed examination on the edge structure as denoted by the red box proved the multilayer character of the graphene sheet (Figure S2B, Supporting Information). In addition, the quantitative analysis in Figure S2C in the Supporting Information revealed that the number of graphene layers ranged from 5 to 12 and the majority of graphene possessed 9–11 layers, reconfirming the characteristic of multilayer graphene sheets.

To test the electromagnetic‐induction capacity of graphene and to clarify whether graphene could transform magnetic energy into electricity, a model of a static graphene/PDMS film under a rotating MF (Figure 1A) was used to mimic the cutting of magnetic lines by graphene. In this study, a permanent magnet rather than a coil was adopted because of its good stability and practicability, particularly the small size required to attain the same MF strength. As shown in Figure 1F, no voltaic changes were observed on the PDMS during the application of the rotating MF. However, a significant alternating voltage (≈1.5 mV) or current (≈±5 to ±10 µA) was detected on the graphene/PDMS film during stimulation of the rotating MF, which is sufficient to promote the neural differentiation of stem cells.^[^ ^42^ ^]^ In addition, the magnitude of the current on graphene varied with the speed of rotation. These results suggested that sufficient electric transformation from magnetic energy could be generated using the graphene film as a mediator based on the electromagnetic induction effect, which was consistent with our hypothesis.

### 2.2 Cytocompatibility Assessment of the Graphene Film

The cytocompatibility of substrates is the basic prerequisite for the subsequent evaluation of neural differentiation. Tissue culture plates (TCP) and PDMS were utilized as two control samples in the following experiments. Primarily, to ensure better attachment of cells to the material, all substrates were treated separately using an oxygen plasma (OP) cleaner. As shown in Figure S3, Supporting Information, PDMS and graphene without OP treatment exhibited strong hydrophobicity. After OP treatment, the contact angle became smaller and the hydrophilicity was enhanced, suggesting that OP treatment increased hydrophilic groups such as hydroxyl and was conducive to cell adhesion.^[^ ^43^ ^]^ Subsequently, cytoskeleton staining, live/dead cellular staining, and the cell counting kit‐8 (CCK‐8) were performed to evaluate the biocompatibility of these substrates for ADMSCs qualitatively and quantitatively (Figure 1G–J). The results of immunofluorescence staining for F‐actin indicated that the ADMSCs on all substrates exhibited typical fibroblast‐like spindle shapes after inoculation for 3 days, and no apparent morphological changes were detected in any of the groups. In addition, the live/dead staining demonstrated that most cells remained viable (Figure 1H); the living cells were stained green using Calcein AM and the dead cells were stained red using propidium iodide (PI). Quantitative analysis revealed that the percentage of surviving ADMSCs cultured on different substrates was greater than 95%, indicating the good biocompatibility of PDMS and the graphene substrate. Furthermore, the CCK‐8 analysis after culturing for 5, 10, and 15 days showed that the viability of ADMSCs attaching to the TCP increased gradually in a time‐dependent manner (Figure 1J). After 5 days of culturing, a slight decrease in the cell population was observed in the cells cultured on PDMS or graphene compared with those cultured on TCP. However, this phenomenon disappeared and even reversed along with prolongation of the culture time (for 10 days), and the cellular viabilities of ADMSCs on PDMS and graphene were similar to those on TCP after 15 days of culturing. These results could be interpreted by the lower adherence ability in the early phase and the excellent proliferative capacity in the later stage. The CCK‐8 measurement suggested that the viability and proliferation were not affected by PDMS or graphene, indicating the good biocompatibility of the substrates. Moreover, the cytocompatibility of PDMS and graphene was also confirmed by the SEM observation of the cells cultured on the different substrates for 3 days, which exhibited the same cellular morphology on all substrates (Figure 1K).

### 2.3 Neural Differentiation of ADMSCs on Graphene‐Based Electromagnetic Effects Driven by Rotating Magnetic Field

**Figure 2:**
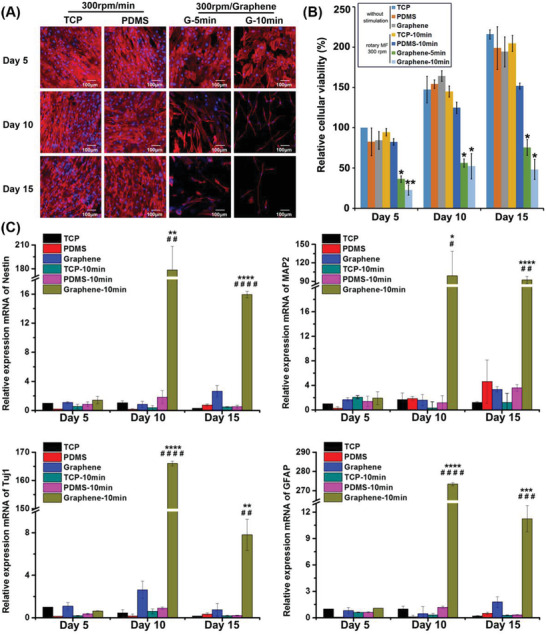
Neural differentiation analysis in cells on graphene film with rotating‐MF treatment. A) Immunofluorescent staining of the cytoskeleton with rotating‐MF treatment. The cytoskeleton was dyed red and the nucleus was stained in blue with DAPI. B) Relative cellular viability of ADMSCs seeded on three substrates under different treatment, which was normalized to those on TCP at day 5. All data represented the mean ± standard deviation (*n* = 3). *, 0.01 < *p* < 0.05; **p < 0.01. C) Relative quantification of neural‐relate genes assayed using q‐PCR. The genes included neural stem cell‐specific gene (nestin), neuron‐specific markers (Tuj1 and MAP2), and marker of astrocytes (GFAP). *p < 0.05; **p < 0.01; ***p < 0.001; ****p < 0.0001, versus TCP group without rotating‐MF treatment. #*p* < 0.05; ##*p* < 0.01; ###*p* < 0.001; ####*p* < 0.0001, versus graphene group without rotating‐MF treatment (*n* = 3).

Previous studies demonstrated that an intermittent current stimulation at 10 µA can induce the neural differentiation of stem cells.^[^ ^44^ ^]^ Based on the results shown in Figure 1F, we selected a 300 rpm rotating MF to treat the cells, which is equivalent to an electrical stimulation of 10 µA. The ADMSCs were seeded on different substrates, and the rotating MF was applied on the top of the cultures at a frequency of 300 rpm for 5 or 10 min each time and twice every day. Cells cultured on different substrates with or without rotating‐MF treatment for 5, 10, and 15 days were collected for cytoskeleton staining. As shown in **Figure** **2**A, cells seeded on TCP and PDMS retained their spindle‐shaped cell morphology even with rotating‐MF treatment after 15 days, which was not significantly different from the cells without MF treatment. In contrast, the cells cultured on graphene under a rotating MF for 5 days exhibited noticeable shrinking cell bodies, and the cells changed their morphology from a fiber‐like shape to a polygonal or rounded morphology. With the prolongation of the rotating‐MF treatment, the body of cells on the graphene film elongated and became thinner, and the axon‐like structure gradually appeared. Moreover, this phenomenon occurred earlier in the group treated with an MF for 10 min each time compared with those for 5 min. These results suggested that the rotating MF itself did not affect the morphology and skeleton of ADMSCs, but the induced electric potential generated on graphene nanosheets driven by the electromagnetic effect changed the fate of the ADMSCs and induced their neural differentiation.

In addition to the changes in cell morphology, the results of cytoskeleton staining indicated that the number of cells in the graphene group treated with a rotating MF was significantly lower, implying the possibility of cell death or blocked cell proliferation during the progression of differentiation. The live/dead staining indicated that under the same rotating‐MF treatment conditions, the cells seeded on graphene/PDMS for 2 days did not exhibit an increased cell mortality rate (Figure S4, Supporting Information), which excluded the possibility of cell deaths. Therefore, we inferred that under the MF treatment, the decrease in cell number was caused by the inhibition of cell proliferation. CCK‐8 analysis was further performed to confirm this speculation. Figure 2B shows that ADMSCs seeded on TCP and PDMS substrates maintained good cell viability under rotating‐MF conditions, and the cell survival rate gradually increased with the extension of culture time. Compared with the groups in which cells were seeded on TCP and PDMS without MF treatment, no apparent difference in cell viability was observed in cells on the rotating‐MF‐driven TCP and PDMS substrates, indicating that the MF treatment did not affect the cell viability of the ADMSCs. However, compared with the control group (cells on graphene without rotating‐MF treatment), MF stimulation resulted in a dramatic downregulation of proliferation in the cells cultured on graphene, and the electric potential induced by the graphene owing to the rotating MF accounted for the decrease in cellular viability. The impaired cell activity was reconciled with an increased tendency of neural differentiation. It is well known that neurogenesis in the central nervous system occurs through rapid progenitor expansion, subsequent loss of proliferative capacity, and finally increasing cell determination. Previous studies demonstrated that neuronal differentiation proceeds cell cycle exit into the quiescent G0 phase^[^ ^45^ ^]^ and declined proliferation appears before neuron fate restriction.^[^ ^46^ ^]^ The results of the present study are consistent with these reports. Since the neuron‐like morphology in cells with rotating‐MF treatment for 10 min twice a day appeared earlier and more typical than that of 5 min twice a day, in subsequent experiments, we adopted rotating‐MF treatment for 10 min twice a day to induce cell differentiation.

To verify that the rotating‐MF‐induced electric potential mediated by graphene can promote the neural differentiation of ADMSCs, we performed quantitative polymerase chain reaction (qPCR) to quantitatively analyze the mRNA expression of genes related to neural differentiation. Nestin, an intermediate filament protein, is widely expressed in neural‐precursor cells^[^ ^47^ ^]^ and is used as a specific marker for NSCs. Figure 2C shows that without rotating‐MF treatment, the mRNA level of nestin in cells on TCP, PDMS, and graphene remained unchanged. In rotating MF, no apparent alteration in nestin expression was detected in cells cultured on TCP and PDMS. Surprisingly, the expression of nestin was upregulated ≈180‐fold in cells that were cultured on graphene using the rotating‐MF treatment for 10 days, which was completely different from ADMSCs cultured on TCP and PDMS under the same culture conditions. The mRNA expression of nestin decreased with further prolongation of culture time. However, on the 15th day, the gene expression of nestin in the cells cultured on graphene under rotating‐MF stimulation was maintained at ≈16 times, which was still significantly higher than that in control groups. This result indicated that the alternating electrical stimulation generated by graphene driven by the rotating MF rapidly enhanced the expression of neural stem cell‐related genes in the early stage, and induced the differentiation of ADMSCs into NSCs.

Notably, the stemness of the ADMSC‐derived NSCs gradually weakened in the later phase of stimulation, suggesting the initiation of neuronal or neuroglial differentiation. In the aspect of neuroglial differentiation, it is well accepted that neural progenitors can differentiate into astrocytes, which can be identified by the specific expression of glial fibrillary acidic protein (GFAP). They can maintain the ion concentration around neurons, participate in the metabolism of neurotransmitters, and have a nutritional and protective function in neurodevelopment.^[^ ^48^ ^]^ The qPCR results of GFAP (Figure 2C) showed no obvious alterations of GFAP expression in the cells cultured on all types of substrates without rotating‐MF stimulation. However, when the rotating MF was applied to graphene, the transcription of GFAP was promoted by ≈275‐fold in 10 days and its level gradually decreased tenfold on the 15th day. Similarly, neuronal class III*β*‐tubulin (Tuj1), a marker of early committed neurons,^[^ ^49^ ^]^ was rarely expressed in the cells cultured on all three substrates without rotating‐MF treatment or on the MF‐stimulated TCP and PDMS groups. However, the mRNA level of Tuj1 was elevated significantly on graphene after exposure to the rotating MF for 10 days (165‐fold higher than the control group), and the trend of enhancement declined to sevenfold on the 15th day, indicating that the electrical stimulation generated by graphene induced ADMSC differentiation into neurons and the ADMSC‐derived neurons became more mature with the prolongation of irritation. This deduction was confirmed by the dynamic mRNA variation in microtubule‐associated protein 2 (MAP2), which was expressed predominantly in the cell body and dendrites of neurons and is widely used as a characteristic marker of mature neurons.^[^ ^50^ ^]^ Remarkably, cells on graphene film with rotating‐MF administration exhibited a stronger expression of MAP2 compared with those in other groups. Moreover, the mRNA level of MAP2 maintained a substantial expression even in the late stage of alternating electrical stimulation (on the 15th day, ≈90‐fold). Another remarkable observation was that the tendency of MAP2 (a mature neuron marker) was completely different from that of Tuj1 (a marker of immature neurons) and GFAP (a specific marker of astrocytes). Considering the decreased expression of GFAP, the long‐term and high‐level maintenance of MAP2 suggested that neurons initiated by alternating electrical stimulation were driven to maturity and implied that mature neurons, but not astrocytes, might be an overwhelming majority in the final phase of neural differentiation.

### 2.4 Wireless Electrical Stimulation Triggering Functional Neural Differentiation of ADMSCs

To further confirm that the wireless electrical signal generated on graphene driven by rotating‐MF conditions induces and favors neuronal differentiation of ADMSCs, we investigated the expression of neural differentiation‐related proteins in ADMSC cultures on different substrates with and without rotating MF stimulation for 5, 10, and 15 days by performing immunofluorescence staining.

**Figure 3:**
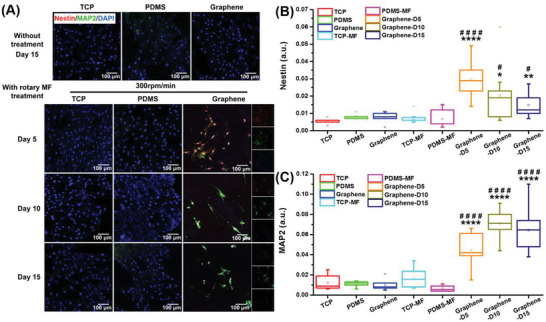
Immunofluorescent staining of nestin and MAP2 in cells cultured on different substrates for 5, 10, and 15 days. A) Images of immunofluorescence staining for nestin (stained red) and MAP2 (dyed green) after treatment for 15 days. Cell nuclei were stained blue with DAPI. B,C) Statistical analysis of the relative fluorescence intensity of nestin and MAP2 in ADMSCs inoculated in different substrates with different treatment. *p < 0.05; **p < 0.01; ****p < 0.0001, versus TCP; #*p* < 0.05; ####*p* < 0.0001, versus graphene group without rotating‐MF treatment (*n* = 4).

The protein levels of nestin and MAP2 in the different samples are shown in **Figure** **3**. As shown in Figure 3A,B, the weak fluorescence of nestin and MAP2 was detected in ADMSCs grown on TCP and PDMS substrates, regardless of the rotating‐MF treatment. Similar fluorescence intensity was also observed in cells cultured on the graphene film. However, when exposed to the rotating MF and stimulated for 5 days with a frequency of 10 min twice a day, cells cultured on graphene substrates exhibited strong fluorescence intensities of nestin and MAP2. With the extension of the stimulation, the expression of nestin gradually decreased on the 10th and 15th day (Figure 3A,B). In contrast, the level of mature neuron marker MAP2 increased continuously and stabilized after 10–15 days of treatment (Figure 3A,C), which was consistent with the qPCR results, indicating that continuous wireless electrical stimulation could promote the neural differentiation and maturation of ADMSCs.

**Figure 4:**
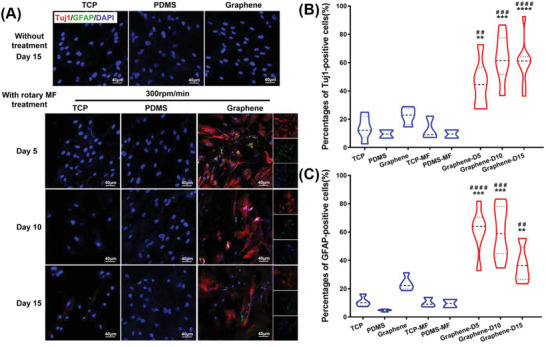
Immunofluorescent staining of Tuj1 and GFAP in cells cultured on different substrates. A) Images of immunofluorescence staining for Tuj1 and GFAP in ADMSCs cultured in different samples with different treatment. Cells were stained with Tuj1 (red) for neurons, GFAP (green) for glial cells, and DAPI (blue) for nuclei. The percentages of Tuj1‐positive cells B) and GFAP‐positive cells C) were calculated and normalized to DAPI in the cell populations. **p < 0.01; ***p < 0.001; ****p < 0.0001, versus TCP group. ##*p* < 0.01; ###*p* < 0.001; ####*p* < 0.0001, versus graphene group without exposure to a rotating MF (*n* = 3).

To clarify the orientation and types of ADMSC‐derived nerve cells induced by graphene‐mediated electrical stimulation, immunofluorescence staining of Tuj1 (an early neuron marker) and GFAP (an astrocyte marker) was performed for the ADMSCs cultured on different substrates. The results in **Figure** **4**A showed that cells in the control group, including TCP, PDMS, graphene, and TCP and PDMS with rotating‐MF treatment exhibited rare expressions of neural or neuroglial proteins. However, Tuj1 and GFAP exhibited high expression levels after rotating‐MF stimulation for 5 days. With the extension of the stimulation, the expression of Tuj1 gradually increased, similar to the tendency of MAP2. Meanwhile, the expression of GFAP gradually increased in the early stage but decreased steeply on the 15th day (Figure S5, Supporting Information). To distinguish the ratio of neurons and astrocytes, the percentages of Tuj1^+^ cells (neuron) and GFAP^+^ cells (astrocytes) were calculated and are shown in Figure 4B,C. The co‐localization fluorescent staining of neurons and glial cells indicated that more glial cells were present in the early stage of differentiation, and the percentage of positive cells was ≈60%, while the rate of neuronal cells was less than 40%. In the late phase of wirelessly electrical‐induced differentiation, the number of neuronal cells gradually increased to 60%, and the percentage of glial cells continued to decrease, which meant that the electrical stimulation generated by graphene was more conducive to the differentiation of MSCs into neurons.

These results proved our hypothesis that because of the promising conductor properties of graphene, induced currents were generated by the cutting of the magnetic induction line when exposed to a rotating MF, and this electrical stimulation without wire connections directly stimulates ADMSCs differentiation into neurons and astrocytes and ultimately results in increased expression of neural differentiation‐related proteins. Meanwhile, the tendency of reduced astrocytes (GFAP^+^ cells) and increased neurons (Tuj1^+^ cells) in the late stage of stimulation suggested a greater proportion of neuronal determination mediated by the graphene‐derived electric potential.

**Figure 5:**
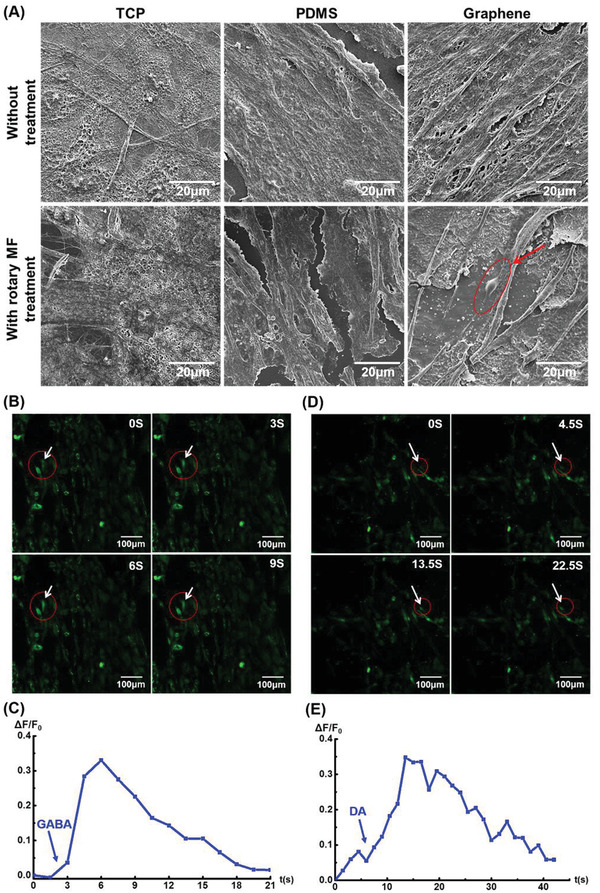
Assessment of typical neuronal morphology and function in ADMSCs‐derived neurons. A) SEM images of ADMSCs cultured in different samples with different processing treatments for 15 days. B,D) Immunofluorescence micrographs to illustrate the responses of cells to neurotransmitters, *γ*‐aminobutyric acid (GABA), and dopamine (DA), in rotating‐MF‐treated cells, respectively (indicated by the arrow). The time (in seconds) after the administration of neurotransmitters is indicated at the top‐right corner of each panel. C,E) Relative intensity of cellular Ca^2+^ per cell in magnetic treatment. The arrows indicate the time when GABA or DA was added. %∆*F*/*F*_0_, changes in fluorescence.

In addition, we performed an in‐depth analysis of ADMSCs cultured on different substrates to verify whether ADMSC‐derived neurons were functional neurons (**Figure** **5**). The SEM image of the cells cultured on graphene after long‐term MF treatment (15 days) exhibited the typical neuron morphology (Figure 5A, indicated by the arrow). In addition, we used calcium ion fluorescent probes to detect whether the stimulation of neurotransmitters could induce calcium sparks, which is the transmission of nerve impulses. This method is widely used to verify the physiological functions of neurons.^[^ ^51^ ^]^ For the cells cultured on TCP and PDMS, no significant calcium sparks were detected, regardless of whether they were subjected to rotating MF. Similarly, with no rotating MF, the cells cultured on graphene did not exhibit calcium influx under the stimulation of neurotransmitters. However, after being cultured on graphene and treated with a rotating MF, a few cells exhibited a transient enhancement of calcium fluorescence accompanied by the administration of different neurotransmitters. As shown in Figure 5B, the fluorescence intensity of calcium was observed at a relatively low level in the cells without the addition of any neurotransmitter, indicating the remaining state of cells. When exposed to an appropriate dose of neurotransmitters such as *γ*‐aminobutyric acid (GABA) or dopamine (DA), the intracellular Ca^2+^ fluorescence increased sharply and briefly in a short period and returned to the resting state level over time. In addition, Videos S1 and S2 in the Supporting Information show that calcium sparks in the adjacent cells appeared sequentially and orientationally after receiving neurotransmitter stimulation, indicating the existence of a network of calcium sparks among cells; in other words, the transmission of nerve impulses. These results indicated that wireless electrical stimulation generated by graphene‐based electromagnetic induction can induce ADMSCs to differentiate into functional neurons, and these ADMSC‐derived neurons can effectively transmit nerve impulses.

Notably, no differentiation‐inducing factors were added to this system. Instead, the electrical potential induced by the rotating MF into graphene was the sole actuator that promoted the neural differentiation of MSCs. The strategy of using the characteristics of nanomaterials to convert external fields into wireless electrical signals avoids the risk of secondary injury and potential infection caused by implanting wires through wired electrical stimulation, and it has a significant value in clinical applications. In an ideal system, autologous ADMSCs seeded on a graphene substrate and transplanted to the nerve injury site can transdifferentiate into functional neurons when a rotating MF with appropriate strength and frequency is applied above the site, in the presence of graphene, which transforms the external MF into wireless alternating electrical stimulation in vivo, and finally repairs nerve damage and recovers neural functions in situ.

Furthermore, the biodegradability of graphene was considered to ensure in vivo biosafety. Previous studies demonstrated that graphene can be biodegraded by human neutrophil myeloperoxidase^[^ ^26^ ^]^ and the degradation products are nongenotoxic to humans.^[^ ^27^ ^]^ In this study, peroxidase diluted in phosphate‐buffered saline (PBS) was used to degrade the graphene film to imitate the microenvironment in vivo. After being subjected to peroxidase for 7 days, Raman spectroscopy and SEM were used to verify the degradation effect of graphene (Figure S6, Supporting Information). The Raman spectroscopy results indicated that the intensity of the 2D peak of graphene treated with peroxidases was higher than that of untreated graphene, and the intensity increased more significantly with the extension of the peroxidase‐treated time, resulting in a gradual increase in the *I*_2D_:*I*_G_ ratio, which implied that the layer number of graphene was attenuated. In addition, the SEM image indicated that the surface of peroxidase‐treated graphene was broken and fragmentary, which was considerably different from the intact appearance in enzyme‐untreated graphene, suggesting the effective degradation ability of peroxidase to graphene. These results provide a guarantee for the in vivo degradability and safety of the graphene substrate used in this study.

Moreover, IR thermography was utilized to measure the possible thermal effect generated during rotating MF stimulation. As shown in Figure S7 in the Supporting Information, the surface temperature of graphene was 20.2 °C without rotating MF treatment. After exposed to rotating MF (300 rpm) for 15 min, the surface temperature increased to 21.2 °C, indicating thermal generation and conduction during stimulation on graphene film. However, this minimal increase in temperature could be dispersed and transported into the culture medium, leading to weaken thermal effect. It had negligible impacts on cell behavior compared with the heat shock at 42 °C, the widely used temperature to interfere neural differentiation. In addition, the stimulation of 10 min twice daily was short and temporary, and the vast majority of cell process was occupied by constant 37 °C. Taken together, the possible effect of thermal potential generated during stimulation on cell differentiation can be ignored.

## 3 Conclusions

In this study, we established a facile and efficient method of inducing autologous ADMSCs to differentiate into functional neurons. This method does not rely on the function of neural‐inducing biological factors, but only utilizes the conducting properties of a scaffold material (graphene) to convert external MFs into electrical fields based on electromagnetic induction. The resultant wireless electric stimulation inspires the neural differentiation potential of ADMSCs and drives them to differentiate into functional neurons to transmit nerve impulses. Compared with the system in which conductive materials connect wires to stimulate neural differentiation, this noninvasive, localized, and in situ electrical stimulation method has potential value in clinical applications for the treatment of neurodegenerative diseases.

## 4 Experimental Section

### Chemicals and Reagents

All chemicals were of analytical reagent grade and were used without further purification. Deionized water was used to synthesize the materials. Iron(III) chloride hexahydrate (FeCl_3_·6H_2_O), hydrochloric acid, and ethanol were purchased from Sinopharm Chemical Reagent Corporation, Ltd. Bovine serum albumin (BSA) and Triton X‐100 were purchased from Solarbio (Beijing, China), and paraformaldehyde solution was purchased from SparkJade (Qingdao, China).

For cell experiments, the ultraCULTURE serum‐free culture medium was purchased from Lonza Bioscience (USA), and the Ultroser G serum substitute was purchased from Pall Corporation (USA). Fetal bovine serum (FBS) was purchased from Gibco (USA). Basic fibroblast growth factor (bFGF) was purchased from Peprotech (USA). Penicillin‐streptomycin solution was purchased from SparkJade (China). The CCK‐8 kit was purchased from MedChemExpress (Shanghai, China), and Trizol reagent was purchased from Invitrogen (USA). The primers for nestin, Tuj1, GFAP, and MAP2 for qPCR were purchased from Sangon Biotech (Shanghai, China). 4’6‐diamidino‐2‐phenylindole (DAPI), nestin, Tuj1, GFAP, and MAP2 primary and secondary antibodies were purchased from Abcam (USA).

### Synthesis and Characterization of Graphene Film

Graphene was grown using thermal chemical vapor deposition.^[^ ^52^ ^]^ A nickel (Ni) plate was used as the catalytic substrate. Briefly, Ni was placed in a split tube furnace and annealed for 10 min at 1028 °C under ambient pressure under argon (Ar) and hydrogen (H_2_) flow. Subsequently, the deposition process was started, in which CH_4_ was introduced into the quartz tube with constant Ar and H_2_ flow for 10 min to obtain complete coverage of graphene (CH_4_:H_2_:Ar = 20:30:270 sccm). After growth, the sample was rapidly cooled to room temperature. The obtained graphene/Ni sample was then dip‐coated with PDMS to protect the carbon layers and immersed in a mixed solution of FeCl_3_·6H_2_O and diluted hydrochloric acid for at least 72 h under constant refreshing of the etchant solution to completely remove the Ni. Thereafter, the graphene film PDMS was washed in deionized water for another 72 h under constant refreshing.

To characterize the substrates, the crystallinity and number of layers in the graphene film were examined using a Raman spectrometer (LabRAM HR Evolution, Horiba, France). The morphology of the graphene film was determined through SEM using a Regulus 8100 (Hitachi, Japan). The surfaces of the samples were determined using AFM (Dimension Icon, Bruker). The composition and structure of the materials were characterized using XPS (AXIS SUPRA, Shimadzu, Japan).

A combination of quiescent samples and a rotating MF was used to mimic the cutting magnetic induction line and to trigger electromagnetic induction, in which the graphene film was immobile and a permanent magnet above substrates spun at 100–300 rpm. The MF intensity generated by magnetic‐irons was ≈0.3 T. The electricity‐generation performance initiated by electromagnetism induction phenomenon was analyzed using a nanovoltmeter (2400, Keithley Instruments Inc., Cleveland, OH, USA).

### Cell Culture and Treatment

The ADMSCs were obtained from the Li Dong laboratory (Qilu Hospital, China) and were cultured in complete culture medium (ultraCULTURE serum‐free culture medium supplemented with Ultroser G serum substitute and 2% FBS) at 37 °C in humidified air with 5% CO_2_. After confluence, the ADMSCs were passaged by 0.25% trypsin and cells in 10‐passage were used for the following experiments.

Before seeding, both the PDMS and graphene/PDMS were cut into pieces with same shape of 2 × 2 cm^2^ and subjected to OP treatment (ATTO, Diener, USA) for 10 min with 100 W of RF power and a 20 cm^3^ min^−1^ oxygen flow to enhance the hydrophilicity and to clean the surface of the materials. Subsequently, the samples were immersed in 75% alcohol overnight and then exposed to UV light for 1 h. After sterilization, the substrates were successively soaked in the primary medium for at least 4 h.

ADMSCs were seeded on TCP, PDMS, or graphene/PDMS substrates. To activate the electrical stimulation initiated by electromagnetic induction, half of the cells on TCP, PDMS, or graphene film were placed directly under the center of a rotating MF for 15 days at a frequency of 5 or 10 min each time, and twice every day at 9:00 am and 9:00 pm. Therefore, cells were divided into six groups: 1) cells cultured on TCP, 2) cells cultured on PDMS, 3) cells cultured on graphene/PDMS (graphene), 4) cells cultured on TCP under a rotating MF (TCP‐MF); 5) cells cultured on PDMS with a rotating‐MF treatment (PDMS‐MF), and 6) cells cultured on graphene/PDMS treated with a rotating MF (graphene‐MF). Cells in all the above groups were maintained in a complete culture medium, without administration of any neurotrophin or neural differentiation‐related inducers during the entire process.

### Cell Viability Assay

Cell Counting kit‐8 (CCK8, MedChemExpress, Shanghai, China) was used following standard protocols described by the manufacturer to quantitatively assess cell activity. Briefly, 1 × 10^4^ ADMSCs were seeded on the substrates in 48‐well plates according to the grouping mentioned above. Cells were treated with or without a rotating MF in a complete culture medium for 5, 10, and 15 days. A 10% v/v CCK‐8 solution per well was added to the medium and cultured for 4 h at 37 °C. The supernatant was transferred to 96‐well plates and the light absorption was measured at a wavelength of 450 nm using a microplate reader (SYNERGY H1, BioTek, USA). Triplicate parallel experiments were conducted and averaged. The viability (%) was expressed as (OD_experimental groups_/OD_TCP group without MF exposure_) × 100%. The viability of cells in TCP was determined to be 100%.

### Live/Dead Staining

Cells seeded on the different substrates were cultured in 48‐well plates for 72 h, and then the culture medium was replaced with a 20 µL serum‐free *α*‐MEM (minimum essential medium) medium containing 4 × 10^−6^ m PI and 2 × 10^−6^ m Calcein AM (Thermo Fisher, USA). Subsequently, the cells were incubated at 37 °C for 20 min. After washing in PBS three times, the ADMSCs were examined under a laser confocal microscope (LSM 800, Zeiss, Germany).

### Cytoskeleton Fluorescent Staining

To visually observe the morphological changes and attachment of cells on different substrates, cytoskeleton fluorescent (F‐actin) staining was performed after the cells were cultured for 5, 10, and 15 days according to the manufacturer's instructions. The cells were fixed in 4% paraformaldehyde, permeabilized with 0.1% Triton X‐100, and blocked with 5% BSA solution at room temperature, followed by washing three times with PBS for 10 min after each step. Subsequently, the fixed cells were incubated using rhodamine phalloidin (Thermo Fisher, USA) for 20 min and then washed with PBS. The nuclei were stained with DAPI for 10 min. The samples were observed under a laser confocal microscope with excitation wavelengths of 594 nm (for F‐actin) and 348 nm (for DAPI).

### Characterization of Cell Morphology

To further observe the morphology of ADMSCs on substrates, 3 × 10^4^ cells were seeded on substrates in 24‐well plates and treated for 15 days. The ADMSCs were then fixed with 2.5% glutaraldehyde overnight, washed three times with PBS, and dehydrated using gradient alcohol (30%, 50%, 70%, 80%, 90%, 95%, 98%, and 100%). After 6 h of freeze‐drying, the samples were sprayed with a layer of Au at a current of 20 µA and observed under SEM.

### Immunofluorescence Staining

ADMSCs (1 × 10^4^ cells) were seeded onto the substrates in 48‐well plates and exposed to a rotating MF for 5, 10, and 15 days. After fixation with 4% paraformaldehyde for 10 min, the cells were permeabilized with 0.1% Triton X‐100 in PBS for 10 min, followed by washing in PBS three times. Samples were then blocked with 5% BSA for 1 h. To evaluate neuronal differentiation, antibodies against neuronal markers were used. These samples were incubated with primary antibodies at 4 °C overnight. Primary antibodies included mouse anti‐nestin (marker of NSCs), mouse anti‐Tuj1, rabbitanti‐MAP2 (neuronal specific markers), and rabbit anti‐GFAP (marker of astrocyte), respectively (Abcam). Next, the samples were washed three times with PBS and then incubated with appropriate secondary antibodies (Alexa Fluor 488 conjugated goat anti‐rabbit or Alexa Fluor 546 conjugated goat anti‐mouse IgG) for 1 h at room temperature. After washing three times in PBS, the cells were stained with DAPI (Invitrogen) for at least 10 min. Fluorescence images were acquired using a laser confocal microscope.

### Real‐Time Polymerase Chain Reaction (RT‐qPCR) Analysis

ADMSCs (1 × 10^5^ cells) were seeded onto the substrates in 6‐well plates. After stimulation for 5, 10, and 15 days, the total RNA was extracted using the Trizol reagent and the mRNA expression levels of nestin, Tuj1, MAP2, and GFAP were analyzed by qPCR. Specifically, cDNA was synthesized from 1 µg of total RNA according to the instructions of the PrimerScript RT reagent kit (Takara, Japan). mRNA analysis was then performed using specific primers for each of the target mRNAs. qPCR reactions were performed using TB Green PCR Master Mix on a Light Cycler 96 Real‐Time PCR System (Roche, USA). *β*‐actin was identified as an internal control and the relative gene expression was calculated using the Δ Ct method.^[^ ^53^ ^]^ Standard cycling conditions were used for all the reactions at a melting temperature of 60 °C. The primer sequences used for real‐time PCR are listed in **Table** **1**.

**Table 1:** Primer sequences

Primer	Forward (5’‐3’)	Reverse (5’‐3’)
*β*‐actin	CTTAGTTGCGTTACACCCTTTCTTG	CTGTCACCTTCACCGTTCCAGTTT
nestin	CACCTGTGCCAGCCTTTCTTA	TTTCCTCCCACCCTGTGTCT
Tuj1	CATGGATGCCGCTCAG	CAGGCAGTCGCAGTTTTCAC
MAP2	CGCTCAGACACCCTTCAGATAAC	AAATCATCCTCGATGGTCACAAC
GFAP	GCAGACCTTCTCCAACCTG	ACTCCTTAATGACCTCTCCATC

### Intracellular Calcium Measurement

Ca^2+^ imaging experiments were performed as described by Ciccolin et al.^[^ ^54^ ^]^ Briefly, the cells were washed with HPBS three times, and 2 × 10^−6^ m Fluo3 (Invitrogen) was added into *α*‐MEM medium to incubate ADMSCs for 30 min at 37 °C. The cells were then washed with Hank's balanced salt solution (HBSS) and fluorescence images (excitation at 488 nm) were monitored using a laser scanning confocal microscope, using image sizes of 256 × 256 pixels and acquisition rates of 0.5–7.5 frames s^−1^. The dopamine solution and GABA solution (0.5 m) were dripped using a 100 µL syringe during the experiment. The fluorescence changes (%∆*F*/*F*_0_) for individual cells were calculated using the formula ∆*F*/*F*_0_ = (*F*_1_−*F*_0_)/*F*_0_ × 100, where *F*_1_ is the fluorescence averaged over the pixel of a cell soma after a stimulus, and *F*_0_ is the average fluorescence of the cell before stimulus application, averaged over three images.

### Statistical Analysis

The data were expressed as the mean ± S.D. from three independent experiments. The statistical significance of the differences was determined using one‐way analysis of variance. For the determination of statistical significance, *p*‐values smaller than 0.05 were considered significant.

## Conflict of Interest

The authors declare no conflict of interest.

## Supporting information

Supporting Information

Supplemental Video 1

Supplemental Video 2

## Data Availability

Research data are not shared.
