# Supplementary material for: Regulation of Neural Differentiation of ADMSCs using Graphene‐Mediated Wireless‐Localized Electrical Signals Driven by Electromagnetic Induction
Source: Adv Sci (Weinh). 2022 Feb 12;9(14):2104424. doi: 10.1002/advs.202104424 (PMC9109060; doi:10.1002/advs.202104424)
Supplement: Supplementary file 1 — Supporting Information [file ADVS-9-2104424-s002.pdf]

ADVANCED SCIENCE
Open Access

Supporting Information

for *Adv. Sci.*, DOI 10.1002/advs.202104424

Regulation of Neural Differentiation of ADMSCs using Graphene-Mediated Wireless-Localized Electrical Signals Driven by Electromagnetic Induction

Zhijie Guo, Chunhui Sun, Hongru Yang, Haoyang Gao, Na Liang, Jian Wang, Shuang Hu, Na Ren, Jinbo Pang, Jingang Wang, Ning Meng*, Lin Han* and Hong Liu**

ADVANCED SCIENCE
Open Access

Supporting Information

for *Adv. Sci.*, DOI: 10.1002/advs.202104424

Regulation of neural differentiation of ADMSCs using graphene-mediated wireless localized electrical signals driven by electromagnetic induction

*Zhijie Guo, Chunhui Sun**,*Hongru Yang, Haoyang Gao, Na Liang, Jian Wang, Shuang Hu, Na Ren, Jinbo Pang, Jingang Wang, Ning Meng**, *Lin Han**, and *Hong Liu**

SupportingInformation

Regulation of neural differentiation of ADMSCs using graphene-mediated wireless localized electrical signals driven by electromagnetic induction

Zhijie Guo¶, Chunhui Sun¶,Hongru Yang¶, Haoyang Gao, Na Liang, Jian Wang, Shuang Hu, Na Ren, Jinbo Pang, Jingang Wang, Ning Meng*, Lin Han*, Hong Liu**

Z. Guo, Associate Prof. C. Sun, H. Gao,N. Liang, J. Wang, S. Hu, Associate Prof. N. Ren, J. Pang, Prof. J. Wang, Prof. H. Liu
Collaborative Innovation Center of Technology and Equipment for Biological Diagnosis and Therapy in Universities of Shandong, Institute for Advanced Interdisciplinary Research (iAIR), University of Jinan, Jinan, 250022, P. R. China
E-mail: ifc_sunch@ujn.edu.cn (C. Sun)
Associate Prof. N. Meng
School of Biological Science and Technology, University of Jinan, Jinan, Shandong, 250022, P. R. China
E-mail: mls_mengn@ujn.edu.cn
H. Yang, Prof. H. Liu
State Key Laboratory of Crystal Materials, Shandong University, Jinan, Shandong, 250100, P. R. China
E-mail: hongliu@sdu.edu.cn (H. Liu)
Prof. L. Han
Institute of Marine Science and Technology. Shandong University, Qingdao, Shandong 266200, China
E-mail: hanlin@sdu.edu.cn

¶Authors contributed equally

Assessment of biodegradation properties of graphene.

Lyophilized HRP type VI (Solarbio, Beijing, China) was solubilized in PBS at 0.385 mg/mL, and then added to the graphene samples into the enzyme solution of 4.0 mL.The graphene was then statically incubated over 24 hours at 4°C in the dark. An excess of 10.0 mL of 80 μM H_2O_2 was added to the bulk sample to start the catalytic biodegradation of the graphene in the presence HRP. Static incubation with H_2O_2(resulting concentration ∼40 μM) was also performed in refrigerated conditions and kept in the dark to avoid enzyme denaturation and photolysis of H_2O_2.

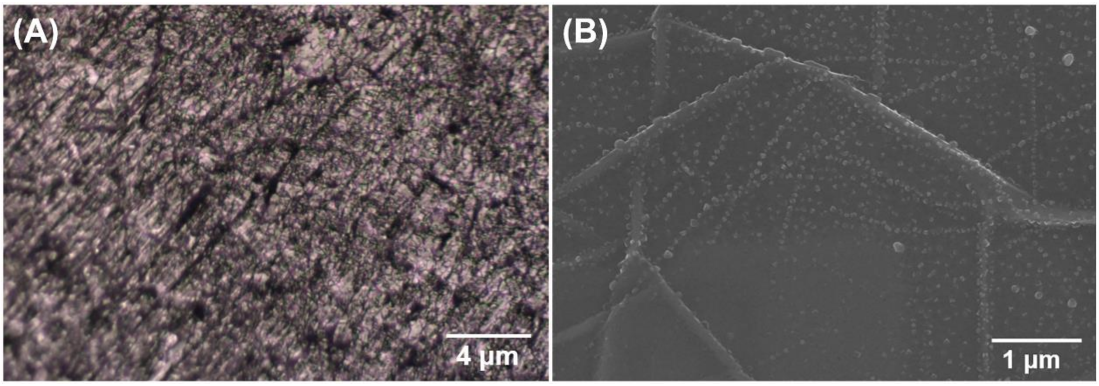

Figure S1.A) Raman image of the substrate (the surface of graphene). B) SEM image of the graphene surface.

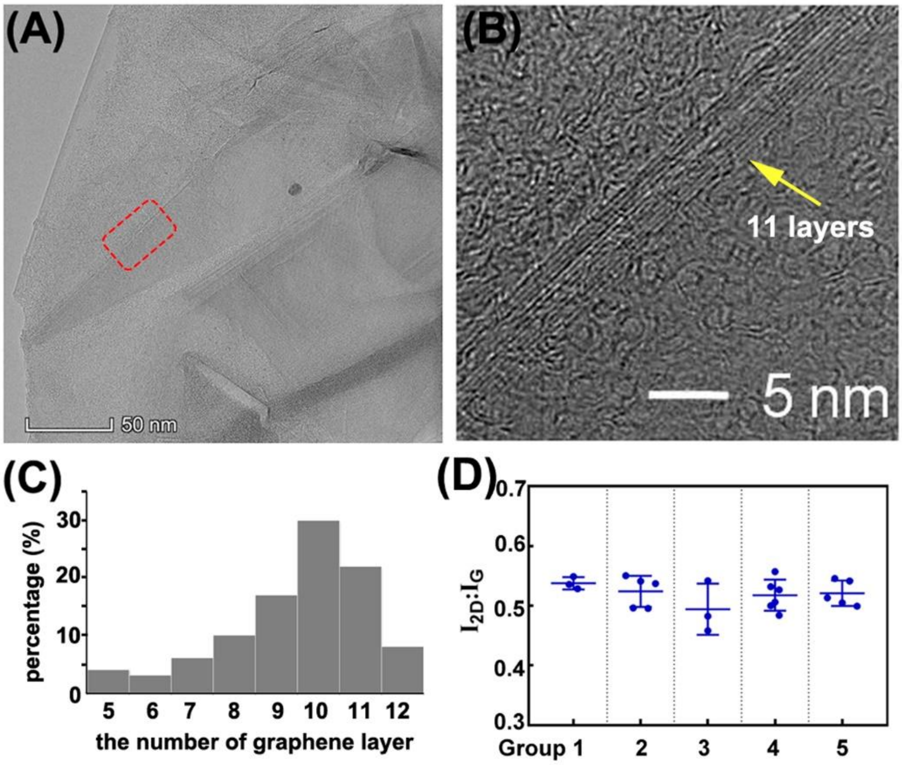

Figure S2. Characterization of graphene layers. A)TEM characterization of graphene layers. B) A magnified edge image obtained from the square-marked area of graphene sheet in panel A. C) The quantification of the number of CVD-synthesized graphene layers. D) Ratio of the Raman 2D- and G-peaks in graphene of different group.

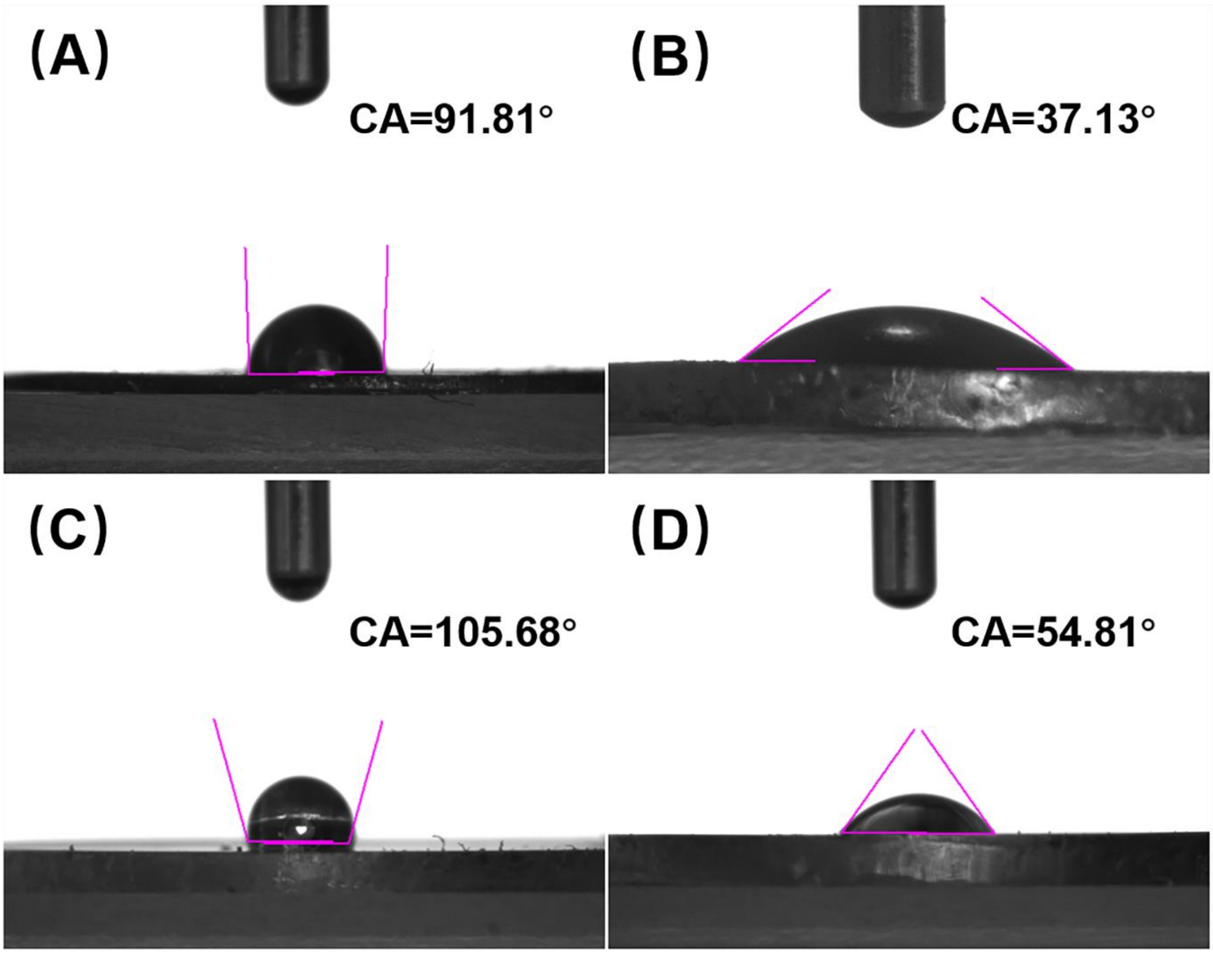

Figure S3. Hydrophilicity of OP-treated graphene. The contact angle images of the A) surface of raw Graphene/PDMS, B) surface of OP-treated Graphene/PDMS, C) surface of PDMS, and D) surface of OP-treated PDMS.

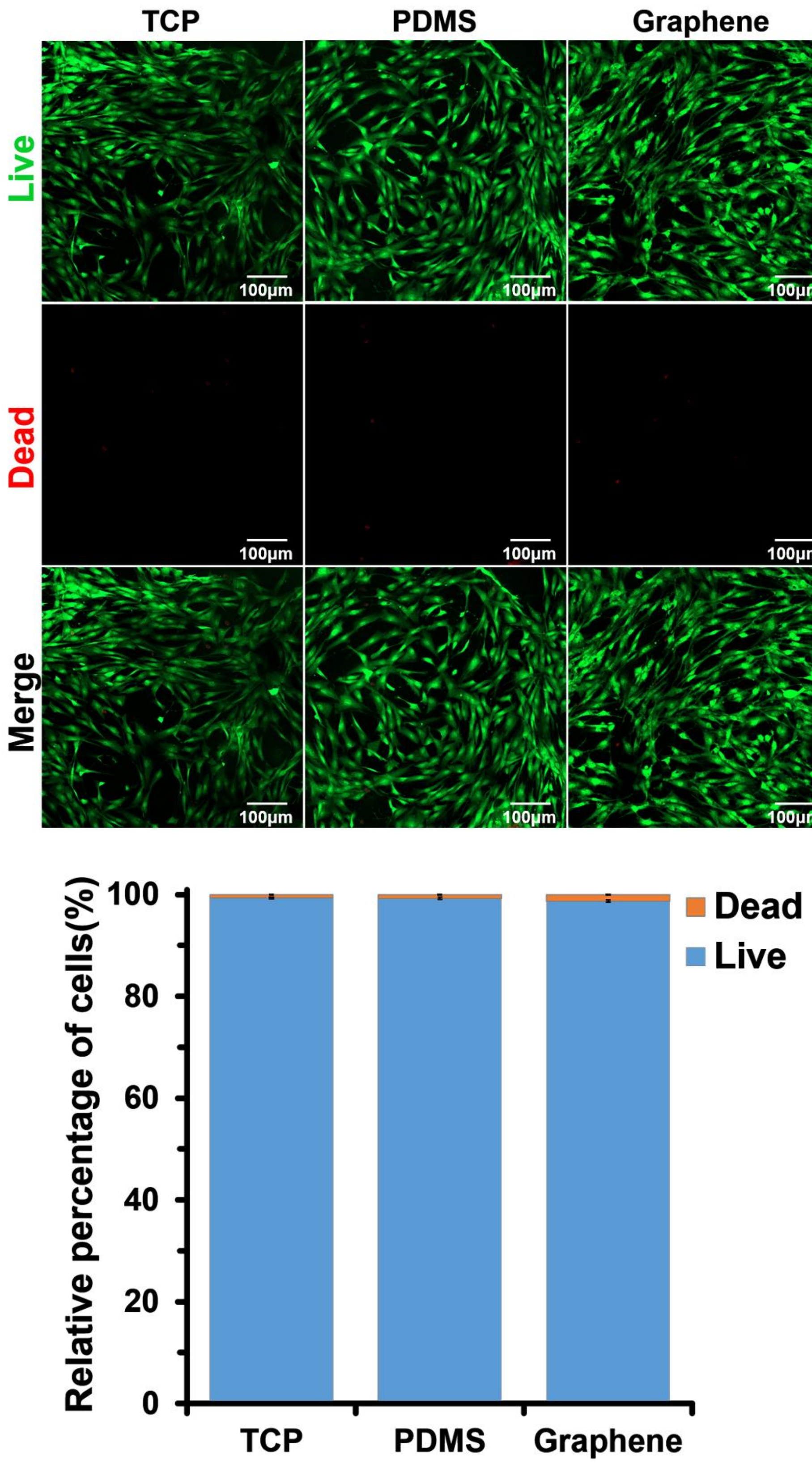

Figure S4. The live/dead staining in different samples with magnetic field treatment. The live cells were stained green, and the dead cells were stained red. Quantification of surviving ADMSCs after culturing in different samples with MF treatment shown in the bottom of image.

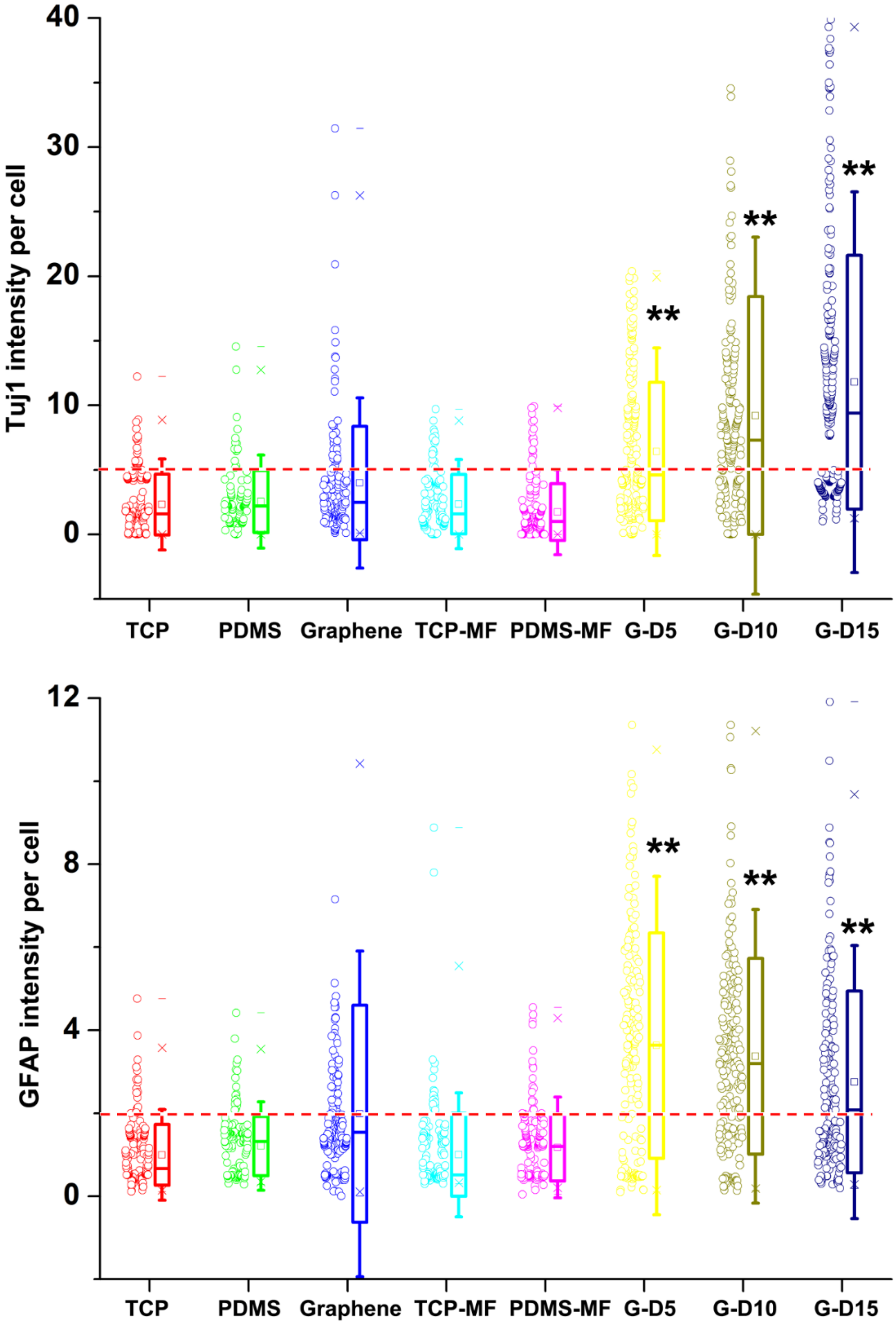

Figure S5. Quantitative analysis of the relative fluorescence intensity of Tuj1 and GFAP in cells on different substrates with different treatment. The boxplots represented the quantification of fluorescent intensity and the bubbles were the individual data of cells. The intensity over the baseline (red dashed line) was deemed as positive cell. ***p*<0.01, vs TCP (n=3).

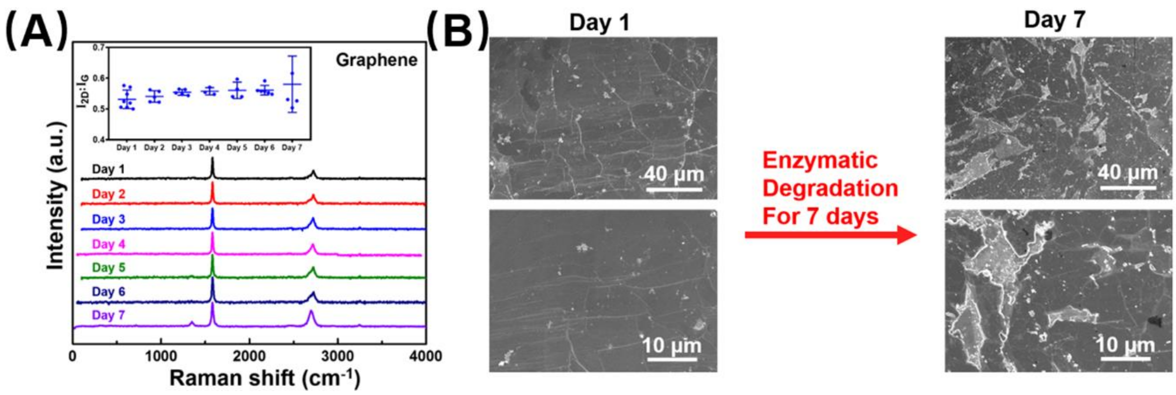

Figure S6. Characterization to monitor the biodegradation of graphene. A) Ratio of the Raman 2D- and G-peaks for the samples at different days; B) SEM images of the surface and the respective magnified SEM images of the same samples at Day 1 and Day 7.

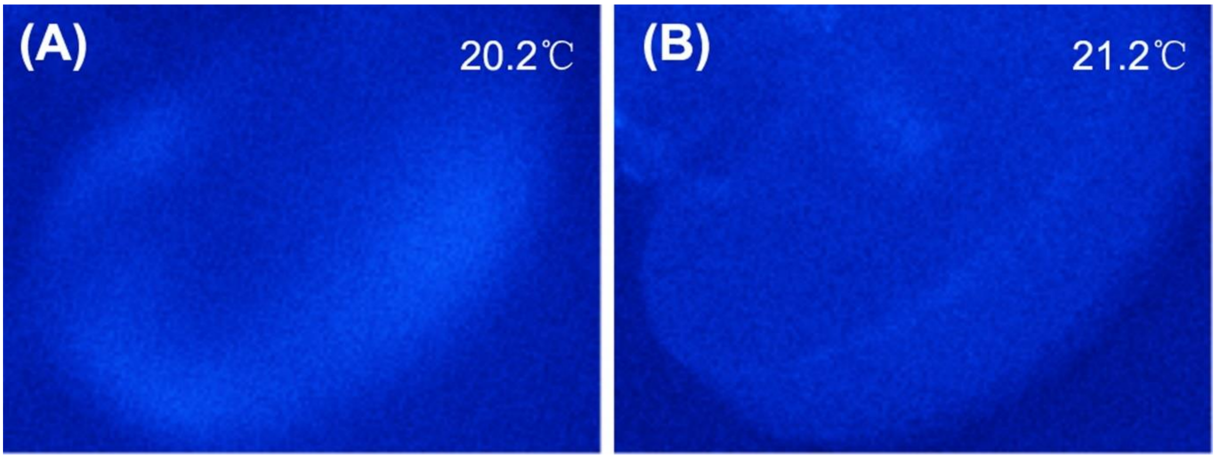

Figure S7. The temperature changes of graphene A) before and B) after rotating MF treatment.

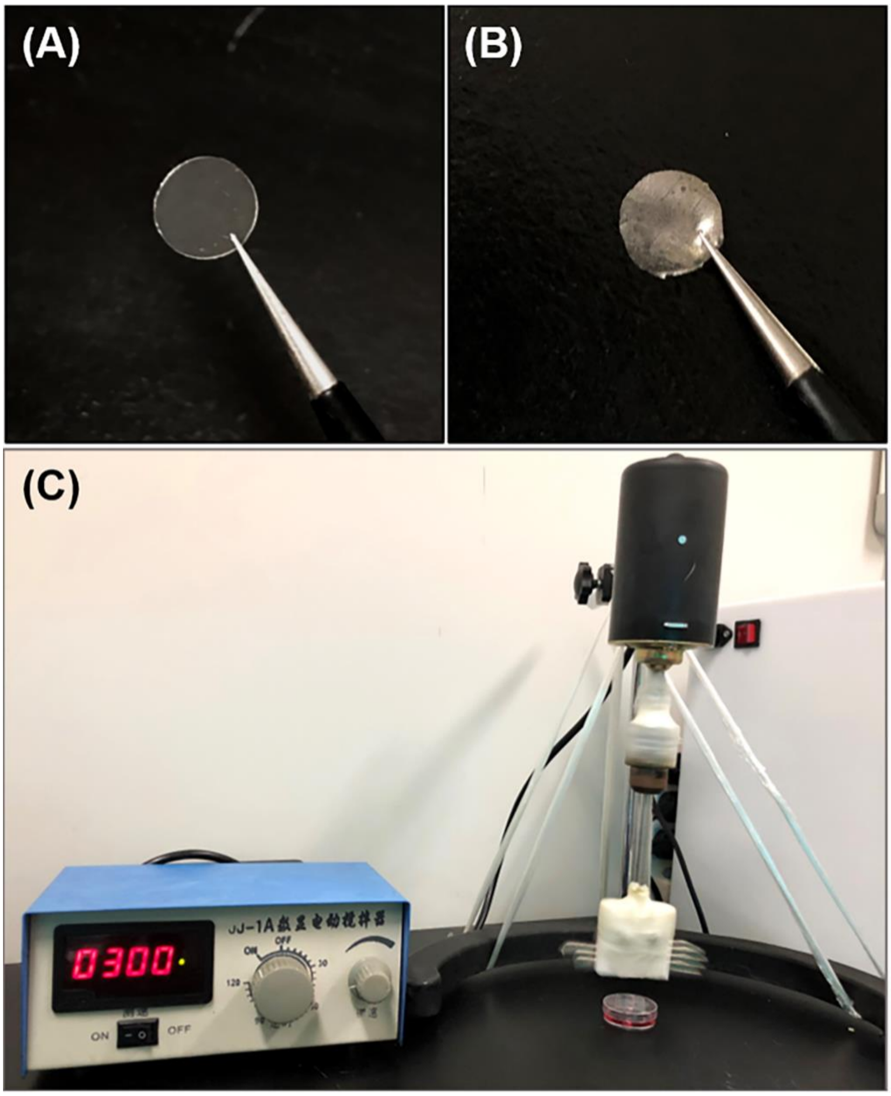

Experimental substrates and equipment. A) and B)The pictures of PDMS and graphene/PDMS substrates used in the experiment. C)The magnetic rotating equipment in the study.

Video 1 Immunofluorescence microscopic video to illustrate the responses of the rotating-MF-treated cells to γ- Reaction (GABA).

Video 2 Immunofluorescence microscopic video to illustrate the responses of the rotating-MF-treated cells to dopamine (DA).
